# Discussion of Dr. Goldsmith’s Paper

**Published:** 1907-08

**Authors:** Marvin H. Smith


### DISCUSSION OF DR. GOLDSMITH'S PAPER.

Dr. Kime: I was very much interested in Dr. Goldsmith's method of operating for hemorrhoids, his manner of dissecting out, suturing, and result attained. I, myself, have been doing a similar operation for ten or twelve years in which the method differs but little. after divulsion of the sphincter, the hemorrhoid is clamped and well turned down, commencing above the hemorrhoid with an over and over, number two, plain catgut suture, trimming off the hemorrhoid with scissors as we go down with the over and over suture so that when hemorrhoid is removed the point is completely sutured, leaving no raw surfaces. If there is puckering of the skin surface, as referred to by Dr. Goldsmith, that portion is caught with forceps and clipped off with scissors and sutured over with the same suture. This work can be done with practically very little loss of blood. I have done it without the loss of over two or three drams of blood. It gives ideal results and since adopting it I have not had any occasion to use any other method. Patients are well in one or two weeks after the operation without leaving any raw surfaces, unless a dressing is used in which the sutures hang and are pulled out when removing the dressing.

Dr. C. R. Andrews: Dr. Goldsmith has mentioned the freedom from infection in the cases upon which he has operated. At first thought, we could be struck by this fact, especially, if one considers the enormous number of bacteria always present in the intestinal contents. This freedom from infection is due, however, to the fact that nominally, there are very few pus-producing organisms in fecal material, and in many instances the feces are entirely free from these organisms. This, I think, explains this freedom from infection mentioned.

In regard to the non-absorption of the chromic suture, used by Dr. Goldsmith, I would suggest that this could be easily obviated by using a cat-gut which has been chromitized by a 1 per cent. chromic aldehyde. By th chromitization for five or six hours in this solution, a gut can be easily obtained, which will become absorbed in about thirteen days.

I described this method of chromitization before the Society a short while ago.

Dr. Willis Jones:—I agree with Dr. Goldsmith, that the operation of excision, is far preferable to any other operation for the radical cure of hemorrhoids and that the operation of ligation should never be done for the simple reason that it only removes that portion of the dilated vein which is protruding and the suffering which the patient has to undergo during the several days while waiting for the ligated mass to sluff away, is sometimes most excruciating and can only be controlled by the administration of large doses of codeine or morphine. After the mass has sloughed away, we cannot be sure that the veins remaining beneath the mucous membrane of the rectum will not at some subsequent day give rise to as serious symtoms as those removed. I most heartily agree with Dr. Goldsmith in making the incision parallel to the long axis of the gut, for serious contractures oftimes follow operations for hemorrhoids where the incision has been made transversely to the long axis of the gut. In the operation of excision, not only the protruding mass of veins can be removed, but also the other dilated veins in the immediate vicinity of the incision can be pulled into the wound, ligated and excised at the same time, thereby lessening the possibilities of future occurrence.

Concerning the clamp and cautery operation, I think it should be limited to those cases where the pile mass is inflamed, because the operation of excision in those cases would not likely be followed by immediate union. The clamp and cautery has the advantage over the operation of excision in that it can be done much more rapidly and there is practically no hemorrhage with the operation, and infection is not so likely to occur. The same principle applies to the clamp and cautery as to the operations for excision, that is, the clamp always should be applied to the long axis of the gut, never transversely, in order to obviate the occurrence of strictures. There are certain advanced cases of hemorrhoids where the operation of excision will not suffice; in this class of cases, the Whitehead or some similar operation will have to be done in order to give the patient the desired relief. As the Whitehead operation is a very bloody one, and since the cut end of the rectum contracts to such an extent that it is sometimes difficult to draw the cut end of the rectum down to the muco-cutaneous junction without making too great tention, several methods have been suggested, one of which is done by Dr. Ellsworth Elliot of New York, who leaves a narrow strip of the rectal mucous membrane anteriorly, posteriorly and laterly, dissecting up the intervening portions, and removing all the dilated veins in the field of operation, igating, excising, then suturing the dissected mucous membrane back into position. This operation has the advantage over the Whitehead, in as much as the hemorrhage is more easily controlled and the tendency to contraction is not so great, since those portions which were left in tact aid in preventing the disconnecting portions from shortening as the lateral edges can be sutured. This operation gives practically the same results as Whitehead's.

After the operation for hemorrhoids, I am a firm advocate of locking the bowels from three to five days in every instance, so as to prevent contamination of the suture line by the fecal contents and prevent the suturing from stretching by the passage of the fecal mass.

Reported By Marvin H. Smith.

